# Idiopathic Tumoral Calcinosis

**DOI:** 10.5334/jbsr.2341

**Published:** 2021-02-09

**Authors:** Jesper Dierickx, Filip Vanhoenacker

**Affiliations:** 1 AZ Sint-Maarten and University (Hospital) Ghent, BE; 2 AZ Sint-Maarten and University (Hospital) Antwerp/Ghent, BE

**Keywords:** computed tomography, magnetic resonance imaging, conventional radiography, tumoral calcinosis

## Abstract

**Teaching Point:** The imaging clues to differentiate idiopathic tumoral calcinosis from other calcified soft tissue lesions include: pseudotumoral appearance with mass effect, bone erosion with intra-osseous protrusion of calcification, lobulated morphology, and peri-articular location on the extensor side.

## Case Presentation

**Figure 1:**
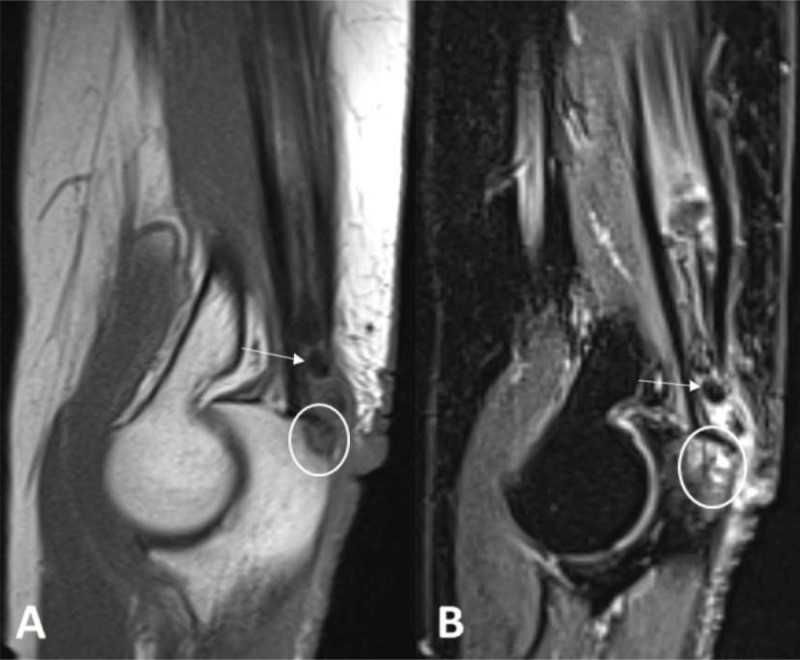


A 68-year-old woman was referred to our department, because of a painful swelling at the extensor side of the right elbow. Magnetic resonance imaging (MRI) revealed an elongated lesion with rosary morphology in the distal triceps muscle belly and tendon. The lesion was predominantly of low signal on all pulse sequences (***Figure 1***, white arrow) surrounded by hyperintense edema on fat-suppressed (FS) T2-weighted images (WI). There was a focal bony lesion on the triceps tendon insertion (***Figure 1***, white circle), hyperintense on FS T2-WI and hypo-intense on T1-WI, though surrounded by a subtle peripheral rim with a lower signal. FS T2-WI demonstrated perilesional bone marrow edema.

**Figure 2:**
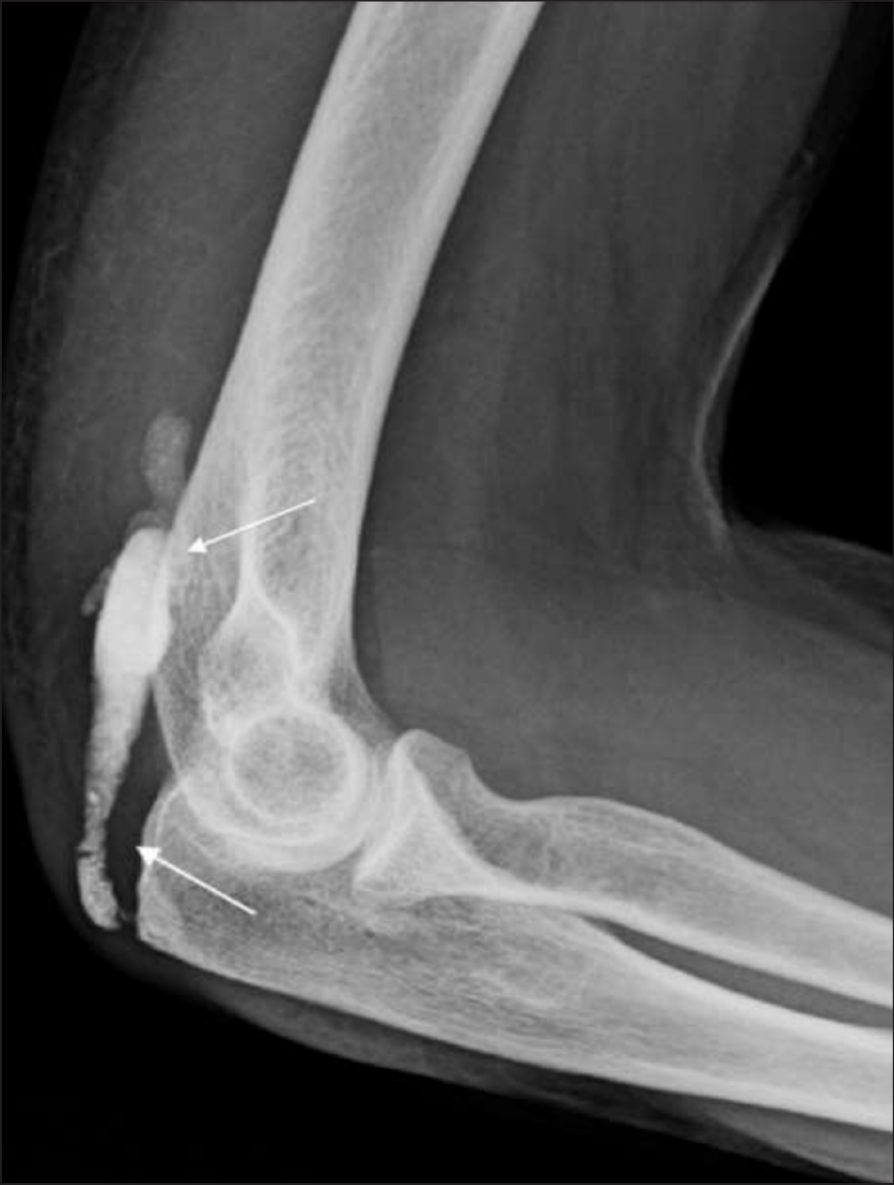


**Figure 3:**
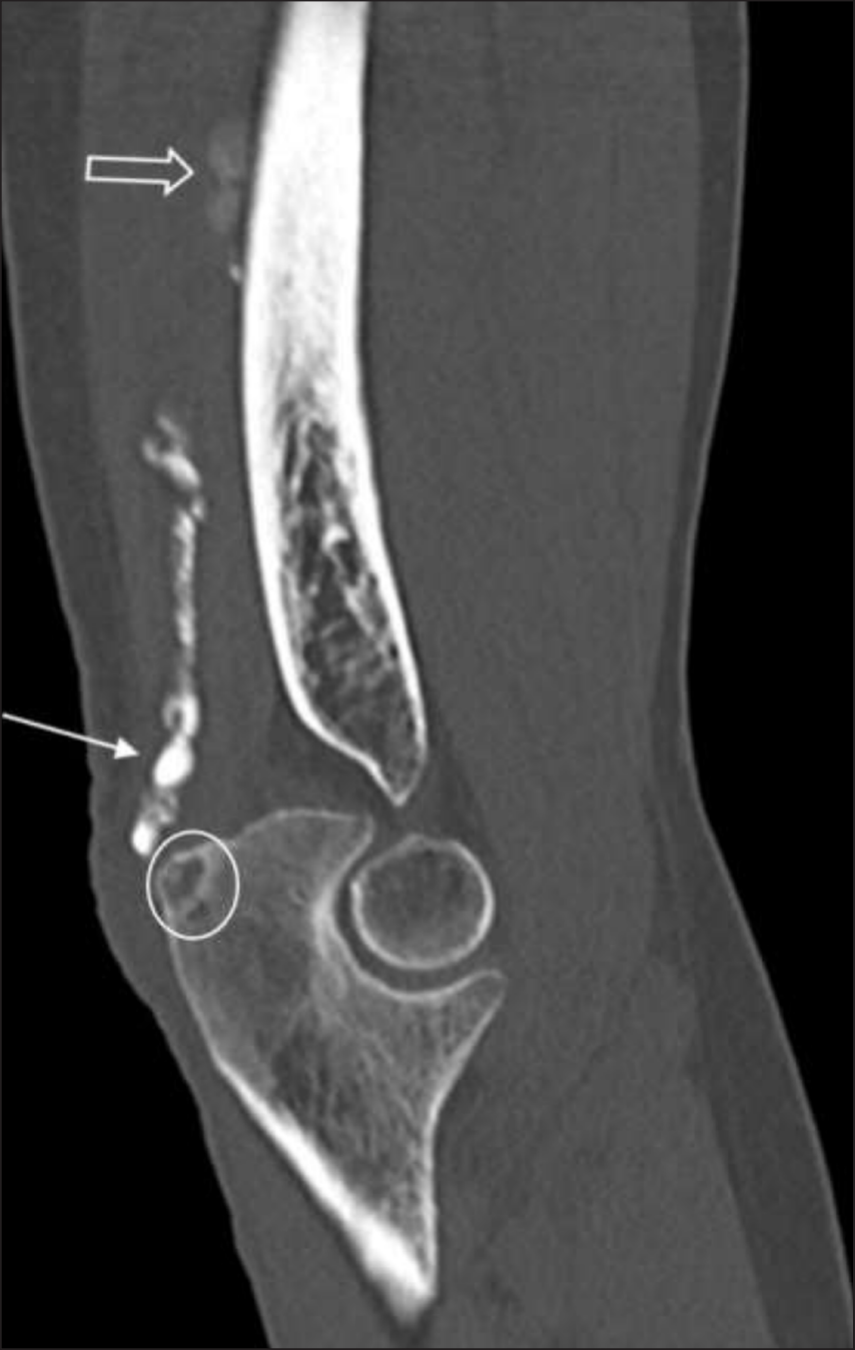


A subsequent conventional radiograph confirmed a well-defined soft tissue calcification with variable density posteriorly to the distal humerus (***Figure 2***, white arrows). Computed tomography (CT) was performed to further evaluate its extent, morphology, and internal structure. CT confirmed an elongated lesion with “string of beads” morphology within the triceps muscle and tendon. The proximal part was less dense (***Figure 3***, void white arrows) than the homogeneously hyperdense distal part (***Figure 3***, white arrows). The olecranon erosion had a subtle central calcification and a sclerotic rim (***Figure 3***, white circle).

Based on the imaging findings, the diagnosis of idiopathic tumoral calcinosis with intra-osseous calcium protrusion was made.

## Comment

Tumoral calcinosis (TC) consists of a peri-articular, calcified soft tissue mass. There is an idiopathic, primary form and a secondary form associated with chronic renal failure and secondary hyperparathyroidism [1]. The disorder occurs typically at the hip, elbow, shoulder, foot or wrist [1]. TC is best evaluated with conventional radiography and CT. Due to its mass effect, potential cortical remodeling, erosion, and intra-osseous protrusion, it may be misinterpreted as a tumoral lesion. The lesion typically has a peri-articular, extensor-sided location. It may be either lobulated and homogeneously hyperdense or have a lower central attenuation with hyperdense walls and potential fluid-calcium levels (“sedimentation sign”). The sedimentation sign and mass effect are typically not present in other calcified pseudotumoral lesions, such as hydroxyapatite deposition, calcific myonecrosis, and myositis ossificans [1].

On MRI, TC is either diffuse hypo-intense on both pulse sequences or heterogeneous with signal voids and areas of high intensity signal on T2-WI, attributed to a granulomatous foreign body reaction [1]. MRI may demonstrate cortical erosion of the adjacent bone with intraosseous migration of calcifications and potential perilesional bone marrow edema on FS T2-WI.

Medical therapy is of limited value in idiopathic TC. Complete surgical excision may be considered in larger lesions [1].
